# Using a Machine Learning Algorithm Integrated with Data De-Noising Techniques to Optimize the Multipoint Sensor Network

**DOI:** 10.3390/s20041070

**Published:** 2020-02-16

**Authors:** Yibeltal Chanie Manie, Jyun-Wei Li, Peng-Chun Peng, Run-Kai Shiu, Ya-Yu Chen, Yuan-Ta Hsu

**Affiliations:** Department of Electro-Optical Engineering, National Taipei University of Technology, Taipei 10608, Taiwan; t105999405@ntut.edu.tw (Y.C.M.); t108658001@ntut.edu.tw (J.-W.L.); t106659002@ntut.edu.tw (R.-K.S.); t107658052@ntut.edu.tw (Y.-Y.C.); t107658053@ntut.edu.tw (Y.-T.H.)

**Keywords:** fiber Bragg grating, wavelength division multiplexing, strain sensing

## Abstract

In this paper, for an intensity wavelength division multiplexing (IWDM)-based multipoint fiber Bragg grating (FBG) sensor network, an effective strain sensing signal measurement method, called a long short-term memory (LSTM) machine learning algorithm, integrated with data de-noising techniques is proposed. These are considered extremely accurate for the prediction of very complex problems. Four ports of an optical coupler with distinct output power ratios of 70%, 60%, 40%, and 30% have been used in the proposed distributed IWDM-based FBG sensor network to connect a number of FBG sensors for strain sensing. In an IWDM-based FBG sensor network, distinct power ratios of coupler ports can contain distinct powers or intensities. However, unstable output power in the sensor system due to random noise, harsh environments, aging of the equipment, or other environmental factors can introduce fluctuations and noise to the spectra of the FBGs, which makes it hard to distinguish the sensing signals of FBGs from the noise signals. As a result, noise reduction and signal processing methods play a significant role in enhancing the capability of strain sensing. Thus, to reduce the noise, to improve the signal-to-noise ratio, and to accurately measure the sensing signal of FBGs, we proposed a long short-term memory (LSTM) deep learning algorithm integrated with discrete waveform transform (DWT) data smoother (de-noising) techniques. The DWT data de-noising methods are important techniques for analyzing and de-noising the sensor signals, and it further improves the strain sensing signal measurement accuracy of the LSTM model. Thus, after de-noising the sensor data, these data are fed into the LSTM model to measure the sensing signal of each FBG. The experimental results prove that the integration of LSTM with the DWT data de-noising technique achieved better sensing signal measurement accuracy, even in noisy data or environments. Therefore, the proposed IWDM-based FBG sensor network can accurately sense the signal of strain, even in bad or noisy environments; can increase the number of FBG sensors multiplexed in the sensor system; and can enhance the capacity of the sensor system.

## 1. Introduction

Due to the primary appealing characteristics of high multiplexing capability, low price, small size, low noise interference, and remote sensing suitability, fiber Bragg grating (FBG) sensors are commonly used for strain, temperature, vibration, and other measurements [1,2,3]. The strain sensing principle of the FBG sensor depends on the shift of the peak wavelength of each FBG due to the change in physical or environmental factors, such as strain, stress, temperature, vibration, pressure, and others [4,5,6,7,8]. In a distributed sensor system, a number of FBG sensors can be multiplexed in the fiber cable using the wavelength division multiplexing (WDM) method. Thus, the distributed FBG sensor system can measure the conditions of different objects in different applications.

However, in a traditional WDM, a unique spectral operational region is assigned to each FBG sensor and the reflection spectra of contiguous FBGs sensors cannot be allowed to overlap. This extremely limits the numbers of FBG sensors in the sensor system. Recently, intensity wavelength division multiplexing (IWDM) techniques have been proposed to improve the multiplexing ability of the sensor system, where the reflection spectra of FBG sensors in the sensor system are allowed to overlap [9]. Thus, IWDM is used to enhance the multiplexing capacity of the FBG sensor network and to enable the sensor system to contain more than twice as many FBGs as traditional WDM techniques [9,10,11]. However, the overlapping spectra of FBG sensors can cause cross talk between the adjacent FBGs and can make it difficult for the traditional wavelength detection techniques to identify the sensing signal of each FBG sensor. Moreover, the FBG sensor system may be influenced by the harsh environment, which affects the shape of the reflection spectra and increases the wavelength detection errors [12].

In real-world applications of sensor systems, the environment is noisy and FBG sensors are vulnerable to the rain and wind. Also, the insignificant vibration makes the spectra of FBG unsmooth, and several burrs occur over the spectrum of FBGs [12,13]. Thus, the noise can degrade the performance and reliability of the FBG sensor system, resulting in large sensing signal measurement errors. As a result, noises from the FBG signal must be filtered using de-noising methods before implementing the sensing signal measurement. Therefore, since the major task in the IWDM-based FBG sensor system is to accurately measure the sensing signals of each FBG sensor from the partially or fully overlapping spectra of FBGs, a correct choice of the interrogation system is critical for the FBG sensor.

Recently, several advanced wavelength detection methods have been proposed for measuring the sensing signal of FBGs from the overlapping spectra. Evolutionary algorithms (EA) are one typical type of method. Recently, several evolutionary algorithms (EA), such as the tree search dynamic multi-swarm particle swarm optimizer (TS-DMS-PSO) [14], genetic algorithms (GA) [15], and the dynamic multi-swarm particle swarm optimizer (DMS-PSO) [16], have been used to enhance the sensing signal measurement accuracy. These EA methods have the ability to accurately detect each FBG’s sensing signal even when the spectra of adjacent sensors are partially overlapped. However, they suffer from a comparatively long processing time in large-structure sensor system.

In addition, there are several types of wavelength prediction algorithms, for example, centroid detection, direct-peak detection (DPD), nonlinear Gaussian fitting, and polynomial fitting [17,18], which are primarily designed to measure the reflection spectrum of a single FBG sensor. However, since, in IWDM-based FBG sensor networks, a number of FBG sensors are multiplexed and there is more than one peak in the reflection spectrum of FBGs, all of the above algorithms cannot work directly in the IWDM FBG sensor systems. Moreover, to improve both the sensing signal measurement accuracy and the speed, machine learning techniques such as extreme learning machine (ELM) and multilayer perceptron (MLP) have been proposed [19,20]. However, the ELM and MLP methods still have less accuracy in the measurements of the sensing signal and increase the time consumption due to the traditional machine learning algorithms having less learning capacity and inflexibility.

Overall, the conventional peak wavelength measurement algorithms listed above have numerous shortcomings, such as experimental noise, sensitivity to power fluctuations, and time consumption. Most recently, in complicated data analysis, deep learning is developing as an advanced machine learning technique. Unlike other traditional machine learning methods, deep learning consists of various hidden layers to learn the features of FBG spectra with various levels of interpretation. Deep learning algorithms can plot the training data to a nonlinear model with a best representation and fitting effect through the multiple hidden layers [21,22,23].

In this paper, a long short-term memory (LSTM) deep learning algorithm integrated with data de-noising techniques is proposed to optimize the performance of IWDM-based multipoint FBG sensor system. LSTM, suggested by Reference [24], is an architectural variation of a recurrent neural network (RNN) that is particularly appropriate for sequences of long input. We applied the LSTM algorithm to recognize and learn the features of the reflection spectrum of FBGs at different strain values and to build the sensing signal measurement model for an IWDM-based FBG sensor network.

The LSTM model can extract wavelength features from the reflection spectra of FBGs. Then, a well-trained LSTM model can measure the sensing signal of each FBG sensor from the overlapping spectra of FBG sensors. Although the LSTM can measure the sensing signal of FBGs, the measurement error may be high due to noises or interference caused by the instability of the broadband erbium-doped fiber (EDF) amplified spontaneous emission (ASE) source and the rough measurement environments. Thus, noise reduction and signal processing play a significant role in strain sensing signal measurements using machine learning techniques.

In this paper, we proposed a DWT data de-noising mechanism in conjunction with an LSTM model to reduce the noise of the sensor dataset and to improve the signal-to-noise ratio prior to training the LSTM model. The experimental results prove that our proposed LSTM model, integrated with discrete waveform transform (DWT) techniques, achieves a better sensing signal measurement of each FBG sensor with a smaller measurement error. Therefore, the contribution of this paper is a data de-noising technique using a DWT function in conjunction with an LSTM model with the aim of providing accurate sensing signal measurements of each FBG and of improving the multiplexing capability and computational speed of IWDM-based FBG sensor systems.

The rest of this paper is structured as follows: The operational principle of our proposed IWDM-based FBG sensor system and the proposed LSTM algorithm is described in Section 2. In Section 3, the experimental setup, data collection, data preprocessing, and design of our proposed LSTM model are presented. In Section 4, the test results and the discussion are presented. Finally, in Section 5, the conclusion is presented.

## 2. Operational Principle of the IWDM-Based FBG Sensor Network

**Figure 1:**
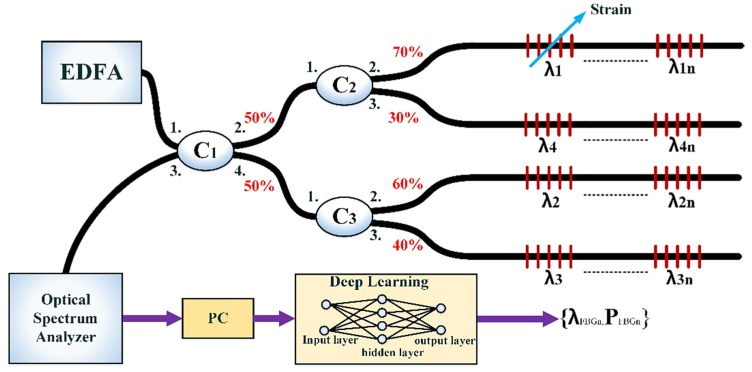
Experimental setup of the proposed intensity wavelength division multiplexing (IWDM)-based fiber Bragg grating (FBG) sensor network. EDFA: erbium doped fiber amplifier, C: optical coupler, PC: personal computer.

Figure 1 shows the experimental setup of the proposed IWDM-based FBG sensor network. The sensor network structure consists of an erbium-doped amplifier (EDFA), optical spectrum analyzer (OSA), optical coupler (C), a personal computer (PC), and FBGs. The EDFA emits the light. The EDFA is utilized to illuminate the FBG sensor array positioned in a parallel structure. The light produced from the EDFA is passed through a coupler (C1), then split into two divisions (i.e., C1 and C2), and fed into FBG sensors. Then, the reflected signals of FBGs are transmitted to the central office (CO) through the C1 coupler. The OSA, located in the CO, can detect and record the reflected spectra of FBGs. Finally, for additional data processing, the detected reflection spectra of the FBG sensors from OSA will be passed to a personal computer (PC). Thus, the PC is used to perform data preparation and to perform the simulation of the deep learning model for measuring the sensing signals of FBGs.

For the proposed IWDM-based FBG sensor system, the total reflection spectra R(λ) of the FBGs is the sum of all FBGs spectra in the sensor system. Assuming that n number of identical FBGs with distinct peak wavelengths are positioned in a parallel structure and that their reflectivity is small enough, the measured spectrum of FBG sensors is expressed as follows:

(1) $$R\left(\lambda ,\lambda_{Bi}\right)=\left(\sum_{i}^{n}R_{i}g_{i}(\lambda ,\lambda_{Bi})\right)+Noise(\lambda )$$

where *λ* is the broadband source’s wavelength, $\lambda_{Bi}$ is the central wavelength of the *i*th FBG, $R_{i}g_{i}$(*λ*, *λ_Bi_*) is the reflected spectrum of the *i*th FBG, *n* is the total number of FBGs, and noise(*λ*) is a random noise.

Furthermore, the return power of the FBGs on the OSA is relative to the broadband source spectrum and the reflection spectrum of the FBGs, which is expressed as follows:

(2) $$I=\int_{0}^{\infty }R(\lambda )Z(\lambda -\lambda_{I})d\lambda$$

where *Z*($\lambda -\lambda_{I}$) is the broadband source and *R*(*λ*) is the spectrum of FBG. Since the broadband source’s spectral width is lower than the FBG bandwidth and the spectrum of FBG is Gaussian shaped, the broadband source’s total returned power can be calculated as follows:

(3) $$I_{i}=C_{i}Z_{\lambda I}R_{m}\exp\left[-4\ln2\times {\left(\frac{\lambda -\lambda_{Bi}}{△\lambda_{B}}\right)}^{2}\right]$$

where $\lambda_{I}$, *R_m_*, $\lambda_{Bi}$, and Δ*λ_B_* are the broadband source wavelength, the peak reflectivity of FBGs, the central wavelength of FBGs, and the FBG’s bandwidth, respectively. *Z* is a delta function, and *C* is the coefficient of power allocation, which depends on numerous influences, including losses of transmission and fluctuations of power. To demonstrate and verify the proposed system experimentally, we use four FBG sensors in this real experiment as we have four FBGs in our lab. Thus, for a four-FBG sensor, there are four Gaussian spectra that return power for each FBG. In IWDM-based FBG sensor systems, the output power of each FBG is different. The reflective spectra of each FBG sensor have a Gaussian shape, calculated by the following:

(4) $$R\left(\lambda ,\lambda_{Bi}\right)=I_{peak}\exp\left[-4\ln2\times {\left(\frac{\lambda -\lambda_{Bi}}{△\lambda_{Bi}}\right)}^{2}\right]$$

where *I_peak_* is the peak reflectivity of the FBGs.

In the proposed IWDM-based FBG sensor system, once the reflection spectra of FBGs enter the overlapping region, the reflection spectra of adjacent FBGs can be overlapped. There are three kinds of overlapping situations, such as nonoverlapping spectra of FBGs, partially overlapping spectra of FBGs, and fully overlapping spectra of FBGs. When the spectra of FBGs are nonoverlapping, the spectra of the four FBGs are separate and the strain sensing signal of each FBG can be easily identified. If the reflection spectra of FBGs are partly overlapped, the strain sensing signal of each FBG may be identified. However, when the reflection spectra of two or three adjacent FBGs are completely overlapped, it is very challenging to measure or identify the exact sensing result of each FBGs from the overlapped spectra using conventional peak detection methods or OSA. Moreover, the partial and fully overlapping spectra of FBG sensors also bring peak wavelength cross talks.

Thus, the conventional peak detection (CPD) techniques cannot understand the overlapped FBG spectra (cross talk) easily and cannot accurately measure the strain sensing signal of each FBG. In this paper, we propose deep learning techniques to overcome the cross talk of overlapping FBG spectra. Our objective is to measure the sensing signal values of FBG1, FBG2, FBG3, and FBG4. However, when FBG1 and FBG2, FBG1 and FBG3, or FBG1 and FBG4 are close and overlap, it is difficult to measure FBG1, FBG2, FBG3, and FBG4 directly from the measured reflection spectrum $R=\left(\lambda ,\lambda_{Bi}\right)$. For this reason, we use the proposed LSTM algorithm to measure the sensing signal of each FBGs. In the model training stage, the reflection spectra sequential feature of the four FBGs at different strain steps is set as the input for training the LSTM model. The training data set is built as follows:

(5) $$D=\left(X_{l},\;Y_{l}\right),\ldots ,\left(X_{k},\;Y_{k}\right),\left(X_{N},\;Y_{N}\right)$$

where $X_{k}R^{l}$ is the reflection spectra FBGs, $Y_{k}$ is the central wavelength of the four FBG sensors at each strain step, and l is the number of sampling points. After completing the training of the LSTM model, the strain sensing signal of each FBG will be measured or recognized from the overlapping spectra of FGB sensors. Thus, the LSTM model can measure the sensing signal of each FBG sensor even if the FBG spectra has cross talk or the overlap problem. The details for the LSTM algorithm are described in the section below.

### 2.1. Long Short-Term Memory (LSTM) Algorithm

**Figure 2:**
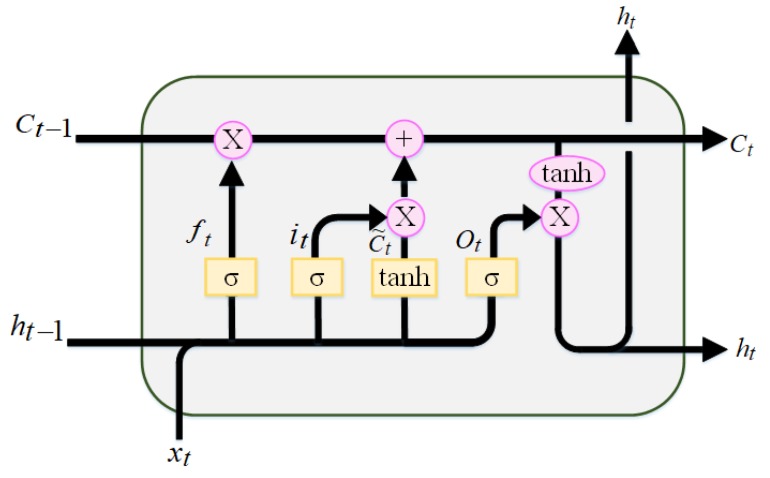
Long short-term memory (LSTM) cell block structure.

The LSTM algorithm is a special kind of recurrent neural network (RNNs) that manages sequential information by memorizing the information for long periods [25,26]. Unlike traditional RNNs, LSTM adds a new framework called a “memory cell” with an internal state to store valuable information [27]. In LSTM, the block of memory replaces the hidden unit structure of the RNN, as shown in Figure 2. The most important structures in the memory block (cell) of LSTM are the three gates and a cell structure. The three gates of LSTM are input gate, output gate, and forget gate. These three gates are applied into the LSTM memory cell of the hidden layer to solve the problem of the vanishing gradient and to thus make it suitable to avoid long-term dependency problems [28,29].

The input gate decides which information in the cell states needs to be updated, and the output gate decides which part of the information in the cell states will be output. The forget gate decides which information should be dropped from the cell state to reset the partial memory [30]. In this way, LSTM has the option of removing or adding information to the cell state rather than fully overwriting cell states as done by standard RNNs [28]. Unlike traditional neural feed-forward networks, LSTM is a sequential algorithm that has a capacity to connect prior information to the current task. Figure 2 shows the LSTM memory cell structure. In the figure, at the time of *t,* the input value to the memory cell is $x_{t}$. The input value helps to capture all sequences of the FBG reflection spectra.

The following equations describe how a memory cell layer is updated at each time, *t.*

First, we calculate the values of the input gate, $i_{t}$, and the values of candidate state, $\tilde{c_{t}}$*,* for the memory cells states at time *t*:

(6) $$i_{t}=\sigma (W_{i}x_{t}+H_{i}h_{t-1}+b_{i})$$

(7) $$\tilde{c_{t}}=\tan h(W_{c}x_{t}+H_{c}h_{t-1}+b_{c})$$

Second, we calculate the activation value of the memory cells of forget gates, $f_{t}$, at time *t:*

(8) $$f_{t}=\sigma (W_{f}x_{f}+H_{f}h_{t-1}+b_{f}).$$

Third, given the activation value of the candidate state, $\tilde{c_{t}\;}$; input gate, $i_{t}$; and the forget gate, $\;f_{t}$, we can calculate the new state, $c_{t}$ memory cells, at time *t*, which is calculated by the combination of $i_{t}$ and $\tilde{c_{t}}$ and of $f_{t}$ and $c_{t-1}$ through the element-wise multiplication (*):

(9) $$c_{t}=f_{t}x\;c_{t-1}+i_{t}x\;\tilde{c_{t}}$$

The final step is to determine the output value. We can calculate the value of the output gates and eventually update the hidden state for the next iteration. Their outputs are calculated as follows:

(10) $$o_{t}=\sigma (W_{o}x_{t}+H_{o}h_{t-1}+b_{o})$$

(11) $$h_{t}=o_{t}x\;\tanh\left(c_{t}\right)$$

$h_{t}$ and $c_{t}$ are transmitted as the input parameters to the next time step, σ represents the sigmoid function between 0 to 1, and the tanh activation function can set the data value within the range −1 to 1.

(12) $$\left(x\right)=\frac{1}{1+e^{-x}}$$

(13) $$\tan h\left(x\right)=\frac{e^{x}-e^{-x}}{e^{x}+e^{-x}}$$

where $W_{i}$, $W_{f}$, $W_{o}$, and$\;W_{c}$ are weight matrices which connect $x_{t}$ to the gates and the candidate value; $H_{i}$, $H_{f}$, $H_{o},$ and $H_{c}$ are weight matrices which connect $h_{t-1}$ to the gates and the candidate value; and $b_{i}$, $b_{f}$, $b_{o},$ and $b_{c}$ are bias vectors of the three gates and candidate value.

**Figure 3:**
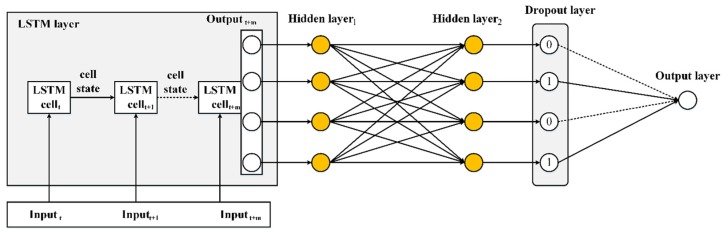
Overview architecture of our proposed LSTM algorithm.

Figure 3 illustrates our proposed architectures of the LSTM algorithms. The first layer of the architecture of our proposed model consists of a layer of LSTM cells. This helps to collect the sensor reflection information about our sensor data throughout different strain sensor values. The LSTM model output may still have remaining nonlinearities; hence, we used two dense hidden layers to solve the distortions or nonlinearities. Then, we implemented a dropout layer to mitigate the risk of overfitting by regularizing the output. Finally, in order to obtain the optimal prediction, the output layer is set to structure the output of the model. Taking the benefit of LSTM in processing sequential data, the sensing signal detection problem is changed into a regression sequential data problem. As shown in Figure 3, the sensing signal detection is considered as a sequential learning problem. When the sequence of reflection spectra of FBGs X = {$x_{1},x_{2},\ldots \ldots ..,x_{m}\}$ is given as an input, an LSTM-based sensing signal measurement model calculates the hidden value $h_{y}$, and then the output value Z = {$\lambda_{BFBG1},\lambda_{BFBG2},\lambda_{BFBG3},\lambda_{BFBG4}\}$ is calculated by the following:

(14) $$h_{y}=\;H(W_{xh}x_{m}+W_{hh}h_{m-1}+b_{h})$$

(15) $$Z=W_{hy}h_{m}+b_{y}$$

where *b* is the bias vector, *W* is the weight matrices, and H(.) is the recurrent function of the hidden layer.

### 2.2. Discrete Waveform Transform (DWT)

DWT is a technique that uses a mother wavelet function to simultaneously analyze a signal in the frequency domain and the time domain. Important data are retrieved from the sensor signal, and noisy data are eliminated from the signal. The wavelet transform will break down signals to low and high frequency to maintain the original data. The wavelet transform breaks down the original signal with processes such as extending and basic wavelet translation. Then, several coefficients of wavelets are obtained. The high-frequency information or low-frequency information of the signals are obtained through high-pass or low-pass filters, respectively [30,31]. Let us say that *n* is the sensor signal length; then the noised signal, *Y*, is expressed as follows:

(16) $$Y_{n}=x_{n}+\;z_{n}$$

where *x* is the important signal and *z* is the unimportant (noisy) signal.

When the noise signal is random and discrete, the resulting wavelet coefficients are therefore relatively low after the DWT. The low-frequency and high-frequency wavelet coefficients are filtered with a pre-seating threshold. The remaining part is then transformed inversely by DWT. Finally, the real signal is constructed. The method of the DWT noise reduction process is described as follows:

i. The original data with noise are collected using Equation (1).
ii. Apply wavelet transform on the data.
iii. Apply threshold processing.
iv. Make it the signal reconstruction.
v. Finally, the noise of the signal is reduced.

After the wavelet transform is applied to the data, to reduce the fluctuations in peak power and shape of the FBG spectra, the threshold λ is expressed as follows:

*λ* = *ε* ⋅ *σ* (17)

where *ε* is the control coefficient and *σ* is the mean square error. *σ* is used as the threshold substrate for wavelet coefficient processing, and *ε* is used as the control coefficient for *σ*. The control coefficient, *ε*, is regulated utilizing the loss established once the training is completed and the threshold *λ* is regulated globally. At last, *λ* can be used to enhance the original wavelet transform techniques. Moreover, since there are different threshold functions, such as hard-threshold de-noising and soft-threshold de-noising functions, the best threshold function can be chosen. The wavelet coefficient (y) is a function of time in terms of the oscillations, which are localized in both time and frequency. In the soft-threshold de-noising method, when the wavelet coefficient |y|<λ, the noise can be reset to zero, while when |y|≥λ, the |y| is subtracted by λ. The soft-threshold de-noising method is expressed as follows [30]:

(18) $$y_{\lambda }=\left\{\begin{array}{c} sign\left(y\right)\left(\left|y\right|-\lambda \right),\;if\;\left|y\right|\geq \lambda \\ 0\;\;\;,\;if\;\left|y\right|<\lambda \end{array}\right\}$$

where *y* is the wavelet coefficient and λ is the threshold. On the other hand, in the hard threshold-de-noising method, when |y|<λ, the noise can be reset to zero and, when |y|≥λ, the wavelet coefficient retains *y* [30]. The hard-threshold de-noising method can be expressed as follows:

(19) $$y_{\lambda }=\left\{\begin{array}{c} y,\;if\;\left|y\right|\geq \lambda \\ 0,\;if\;\left|y\right|<\lambda \end{array}\right\}$$

The final step is that the signal is obtained by the inverse wavelet transform to reconstruct the real signal and to eliminate the noise from the signal.

## 3. Experimental Setup and Data Collection

### 3.1. Experimental Setup

The experimental setup of the IWDM-based FBG sensor system is presented in Figure 1. The output power of the EDFA light source is approximately 16 dBm. The four ports of an optical coupler, with distinct output power ratios of 70%, 60%, 40%, and 30%, have been used in the proposed distributed IWDM-based FBG sensor network to connect a number of FBG sensors for strain sensing. An IWDM technique is suggested to enhance the multiplexing capacity. The EDFA broadband source was used in the FBG sensor scheme to illuminate the four FBG sensors. The central wavelengths of FBG1, FBG2, FBG3, and FBG4 are 1542.34, 1545.2039, 1545.5393, and 1545.8403, respectively. The full width half maxima (FWHM) of the four FBG sensors is 0.2 nm, and the resolution of OSA was set to 0.1 pm. The span width of the OSA was set to 5 nm and was sampled by 2001 points. In our proposed experimental setup, the number of FBGs sensors increases four times the traditional WDM, as each output path of the coupler (i.e., four paths) can support a number of FBGs. The details regarding how the training data were collected are discussed below:

### 3.2. Data Collection and Preprocessing

**Figure 4:**
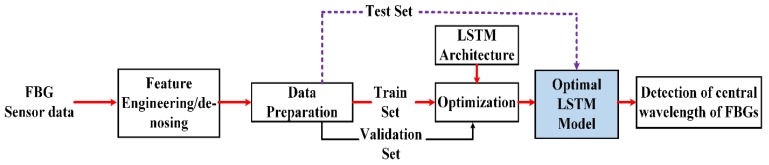
Training procedures.

Figure 4 shows the data processing and preparation process. As for the data preparation, the main objective is to learn the direct correlation between the strain and the reflection spectrum of FBG. During the experiment, the first step is to record the spectra of FBG sensors based on applying a distinct strain to the FBG1 sensor. The experiment is conducted based on the setup shown in Figure 1. For training the LSTM model, the training dataset and testing dataset are recorded using Equation (1), with specified parameters in the experimental setup, including the peak power of each FBG, FWHM, central wavelength of each FBGs, and sample points. The training and testing dataset are the reflection spectra of four FBGs at distinct strain values of FBG1. Thus, we collect a number of training datasets by applying different strain values to the FBG1 sensor (i.e., 0–1285 με) until we find the maximum strain value. When strain is applied to FBG1, the central wavelength of the FBG1 sensor is shifted in the range from 1542.34 nm to 1547.34 nm and the measured spectra of FBGs are sampled by 2001 points, whereas the central wavelengths of other FBGs remain fixed. The strain applied to FBG1 sensor at each strain step is ~41 με.

**Figure 5:**
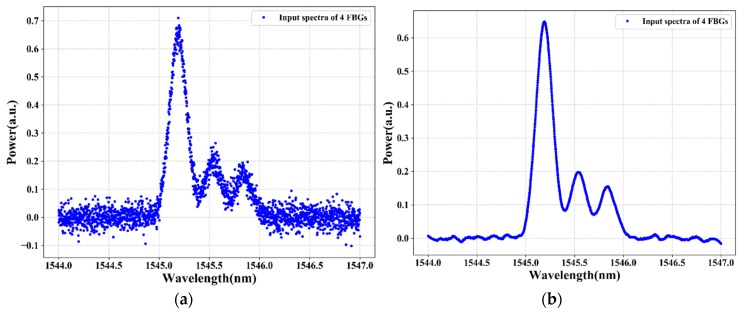
The spectra of four FBGs when two FBGs overlap: (**a**) before applying discrete waveform transform (DWT) and (**b**) after applying DWT.

**Figure 6:**
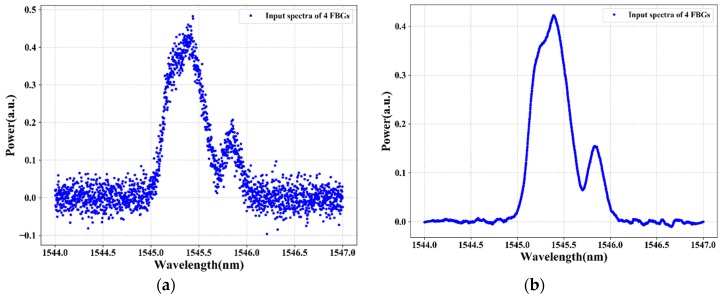
The spectra of four FBGs when three FBGs overlap: (**a**) before applying DWT and (**b**) after applying DWT.

Figure 5 and Figure 6 illustrate the reflection spectra of the FBG sensors at FBG1 stain values of 335 με and 595 με, respectively. As shown in the figures, the spectra of two or three adjacent FBGs overlap fully or partially. The training and testing data samples are recorded using an optical spectrum analyzer by changing the strain applied to FBG1. The collected training data (i.e., the four FBGs reflection spectra at distinct strains) are denoted as *z* = [z1, z2…zi], where *i* is the length of the data input dimensionality. We have 2001 total features as input dimensionality to the proposed system. However, the FBG sensing signals are subject to unstable reflection spectra and noises in practical applications due to the instability of the output power, harsh environment, random noise, deviations in the shape of reflection spectra, aging of the equipment, and other environmental factors that affect the reflection spectra shape, which makes it difficult to distinguish the sensing signals of FBGs from the noise spectrum or noise data.

The reflection spectra features, such as asymmetry, spectral broadening, and top fluctuation, can reduce the sensing signal measurement accuracy. As a result, to adapt and prove our proposed system even in bad environments or noisy data, we train and test the proposed model using noisy data. Due to white Gaussian noise better simulating random noise when the cause of the noise is very complicated, we add white Gaussian noise to the original sensor data using Equation (1). Figure 5a and Figure 6a show the spectra of four FBGs after random noise is added in the original sensor data. The signal-to-noise ratio (SNR) of the noisy FBG signals is 20 dB. However, due to the presence of a high level of noise in the FBG sensor data, the LSTM deep learning model cannot accurately measure the sensing signal of each FBG. To solve this problem, in this paper, the DWT de-noising method is proposed to reduce the noise of the training data (noisy sensor data). The data de-noising process using a wavelet transform of the hard/soft threshold technique can eliminate the noise from the signal.

As shown in Figure 5b and Figure 6b, the noise of the sensing signal is filtered and the spectra of the FBGs look clear. Among the number of spectra of FBGs that are used as training data (output data) that feed into our proposed algorithm, Figure 5b and Figure 6b show the spectra of four FBGs at strain values of 335 με and 595 με, respectively. Then, the data after DWT de-noising is used as the training data for the LSTM model. Thus, after the data de-noising and data preprocessed procedure of the sensors data is completed, the sensor data are fed into the LSTM model to measure the sensing signal of each FBG. The LSTM can learn and understand the features from the reflection spectra of FBG sensors and can design the sensing signals measurement model for the FBG sensor system.

The preprocessed dataset has been divided into training and testing datasets, as shown in Figure 4. Each has a similar number of features and target values. Before training, the training data are normalized to [0, 1] using min-max scaling. The sequence of the reflection spectra data of four FBGs at different strain steps is used as the input data, and the central wavelengths of FBG1, FBG2, FBG3, and FBG4 at different strain steps are used as target data for training the LSTM model. The training data are used to train the network and to adjust the parameters iteratively to minimize the loss function of the model. Then, the well-trained LSTM model estimates the sensing signal of the unknown test samples from the test data, and a test loss is measured. Therefore, the sensing signal measurement of FBGs can be rapidly determined by sequentially feeding the reflection spectra of FBGs into the well-strained LSTM model.

## 4. Results and Discussion

### 4.1. Determining the Optimal LSTM Parameters

**Figure 7:**
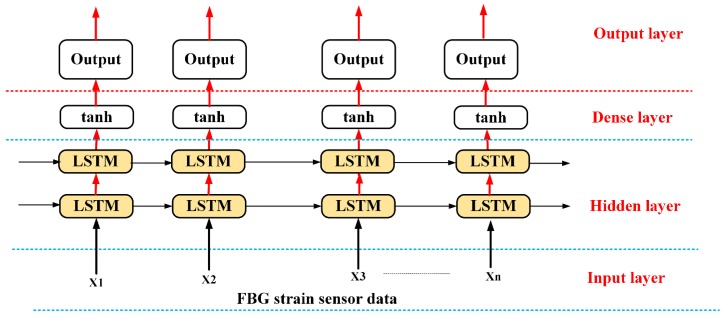
The flowchart and training process of the proposed LSTM model.

Our proposed LSTM model is implemented using the TensorFlow framework, in conjunction with the Keras and Sklearn libraries. The simulation part of this paper runs on a PC, which has an Intel Core i7-4790 3.60 GHz GPU and 20.0 GB RAM. Figure 7 shows the flowchart and training process of the proposed LSTM with four hidden layers and two fully connected layers. The basic architectural structure of our designed LSTM network is as follows: First, the collected training dataset (strain sensing signals) is preprocessed, removes the noise using data de-nosing techniques, and is structured according to the machine training formats. Then, to train the LSTM algorithm, the preprocessed reflection spectra of the FBGs are used as inputs to the LSTM and the corresponding peak wavelengths of FBGs are used as target values. We adjust the different parameters, such as epochs, hidden layers, batch sizes, and optimizer and activation functions, until optimal values are obtained. Then, the well-developed LSTM model is tested by using unseen test datasets. Finally, the prediction outputs are generated by the dense layer, and then, we use a loss function to compare the prediction outputs with the actual values. The prediction performance of our proposed model is evaluated through root mean square error (RMSE).

**Figure 8:**
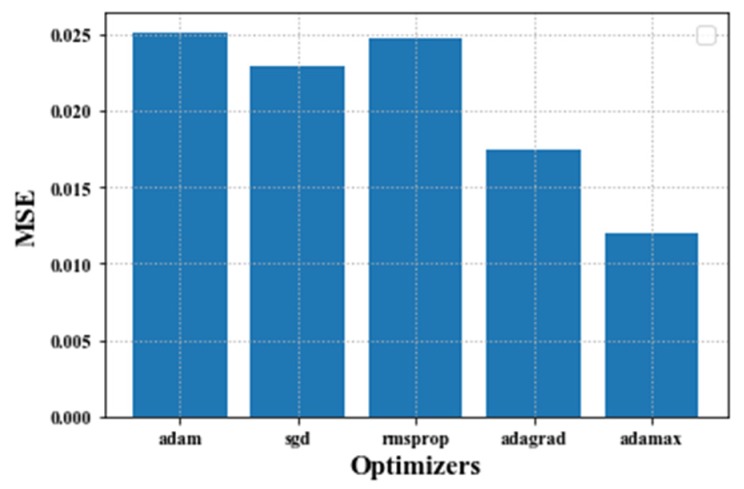
The mean square error (MSE) performance of difference optimizers.

During the training of the LSTM, various parameters such as the number of epochs, batch sizes, hidden layers, hidden units, and optimizer and activation functions are adjusted until optimal values are obtained. Tuning all these parameters results in different training times, root mean square errors (RMSE), and mean square errors (MSE). To know the optimal optimizers, we train the model with distinct optimizers [32] and compare the performance based on MSE (see Figure 8).

As illustrated in Figure 8, Adamax achieves a smaller MSE (i.e., 0.025 pm) than the other optimizers, as adamax is computationally efficient and minimizes noise. Thus, the proposed algorithm is trained using the Adamax optimizer. We also use different activation functions [33]—sigmoid, Relu, tanh, and softmax—to squash the output of the proposed algorithm and to compare the performance. Moreover, we apply dropout regularization within the LSTM layer.

Throughout the training period, a portion of the input units are dropped randomly at each update, both at the input gates and at the recurrent connections, resulting in a lower probability of overfitting and a better generalization performance. Hence, several trainings have been computed on the proposed algorithm until we found the optimal outputs. Therefore, after several trainings, we compile the network using the Adamax optimization algorithm and tanh activation function. Finally, the prediction outputs are generated by the dense layer, and then, we use a loss function to compare the prediction outputs with the actual values. The loss metrics for evaluating the training and validation losses of the proposed model is MSE. The MSE can be calculated as follows:

(20) $$\mathrm{MSE}=\frac{1}{n}\sum_{i=1}^{n}{\left(y_{i}-\bar{y}\right)}^{2}$$

where *n*, $y_{i},$ and $\bar{y}$ are the number of predicted values, the actual value, and the predicted value, respectively.

**Figure 9:**
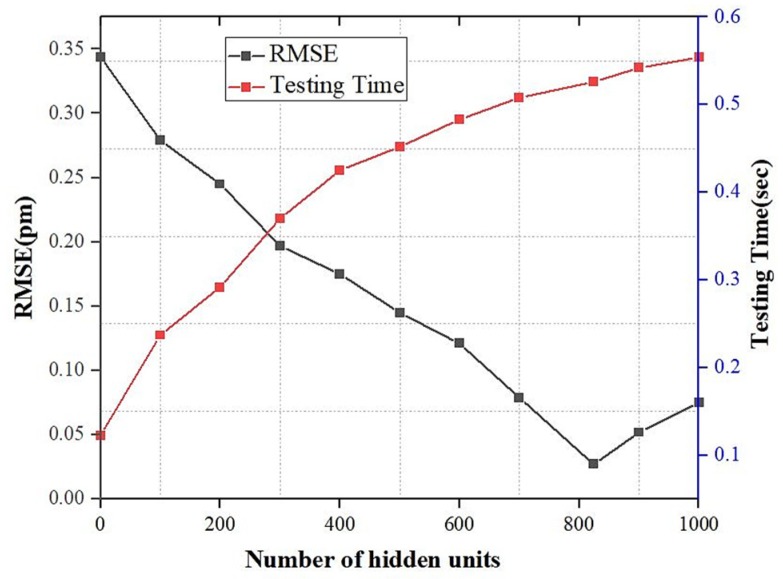
The performance of our proposed LSTM model in terms of root mean square error (RMSE) and testing time with distinct hidden units.

Moreover, to obtain the optimal model, we trained the LSTM model with a variety of hidden units to choose the best number of hidden units. The comparison of the LSTM models’ RMSE with various hidden unit numbers is shown in Figure 9. As shown in the figure, the RMS error decreases as the numbers of hidden units increases. However, when the number of hidden units increases, this requires more testing time. The number of hidden units is saturated, and the error increases after the 824th hidden unit. The maximum test time for the 824 hidden unit LSTM model is 0.526 s. Therefore, for the proposed LSTM model, the optimal hidden unit’s number is 824, which achieves both acceptable accuracy and test time.

**Figure 10:**
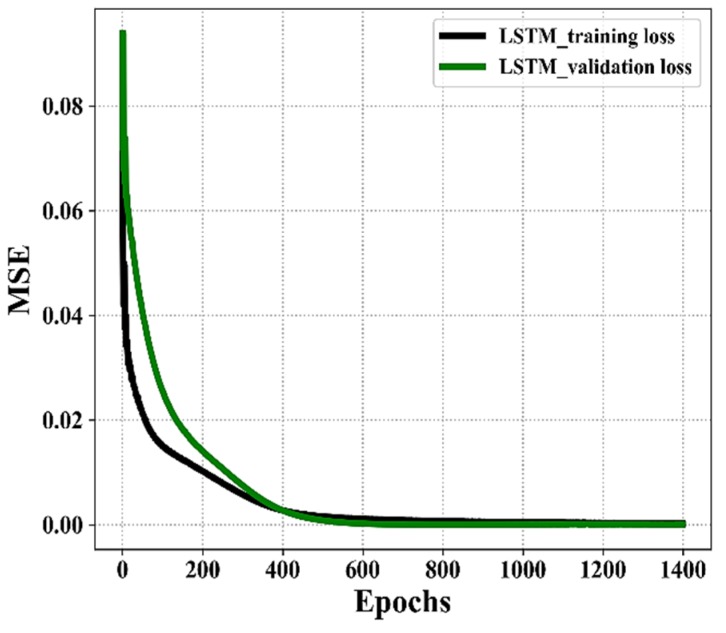
Training losses and validation loss Performance during the training process of the LSTM models.

Moreover, Figure 10 shows the MSE variation of the training and validation losses of our proposed LSTM model with various epoch numbers. Both the training and validation losses reduce significantly with the increase of the epochs/iterations. When the epoch number exceeds 250, the training loss reduces slowly and the validation loss varies in the range of 0 pm to 0.014 pm. The training loss and validation loss converge quickly after approximately 800 iterations, while the optimum value is obtained at the 1400th iteration. The training and validation losses of LSTM are 0.003 and 0.0005 pm, respectively.

**Figure 11:**
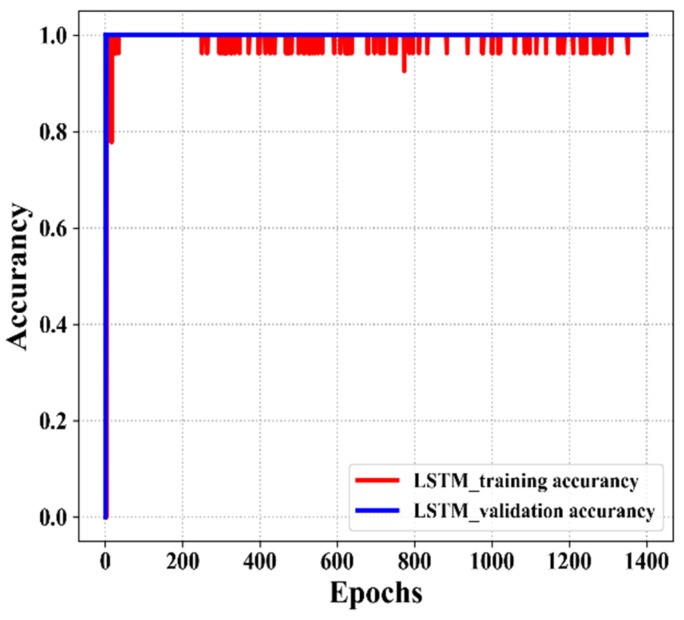
The training accuracy and validation accuracy of the LSTM models.

Figure 11 shows the training accuracy and validation accuracy of our proposed LSTM models with different epoch numbers. As shown in the figure, when the epoch number increases, the accuracy of the model also increases. Thus, at epoch 1400, the LSTM validation accuracy (blue color) achieves 100% accuracy and the LSTM training accuracy (red color) achieves 95% accuracy. Therefore, after several training computations, the optimal values are achieved using the following parameters: 824 hidden units, 1500 batch size, 1400 epochs, and four hidden layers for the well-trained LSTM model, which achieves an acceptable accuracy and testing time.

### 4.2. Model Testing

**Figure 12:**
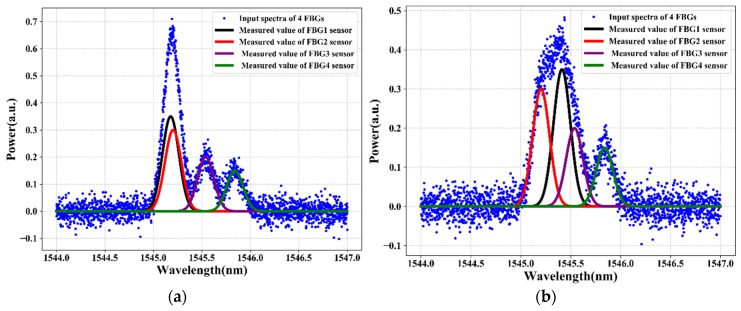
The output results of LSTM in four testing cases without using the DWT smoothing function (**a**) when two FBGs are overlapped and (**b**) when three FBGs are overlapped.

**Figure 13:**
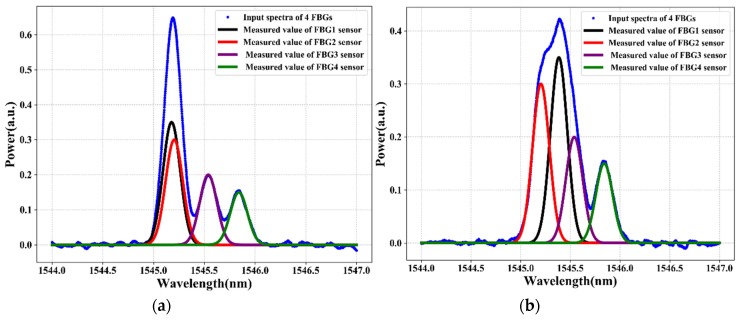
The output results of LSTM in four testing cases using the DWT smoothing function (**a**) when two FBGs are overlapped and (**b**) when three FBGs are overlapped.

This section describes the strain sensing signal measurement performance of our proposed LSTM deep learning model. To test the strain sensing signal measurement performance of the well-trained LSTM, we have taken the unseen test data (reflection spectra of FBGs) from OSA. To test the sensing signal measurement performance of our well-trained LSTM model, we use four different testing cases. Thus, the test data focus on when the situation of the spectra of two or three adjacent FBG sensors are partially or fully overlapped (see blue color spectrum in Figure 12 and Figure 13). As shown in Figure 12 and Figure 13, when two or three FBG sensors are overlapped, the output power (intensity) is the sum of the two or three sensors and the peak power is high. To test the strain sensing signal measurement accuracy performance of our proposed model, we use *RMSE* evaluation methods, defined as follows:

(21) $$RMSE=\sqrt{\frac{\sum_{i=1}^{n}{\left(y_{i}-\bar{y}\right)}^{2}}{n}}\;$$

where *n*, $y_{i},$ and $\bar{y}$ are the number of predicted values, the actual value, and the predicted value, respectively.

Therefore, the sensing signal measurement of FBGs can be rapidly determined by sequentially feeding the reflection spectra of FBGs into the well-strained LSTM model. As a result, the strain sensing signals output the results of the well-trained LSTM model for four distinctive test cases, as shown in Figure 12 and Figure 13. Figure 12a,b shows the sensing signal measurement output of our proposed LSTM model without using DWT data de-noising techniques when the spectra of two FBGs are overlapped (see Figure 12a) and of three FBGs are overlapped (see Figure 12b). As shown in Figure 12a, the proposed LSTM model can measure the sensing signals of FBGs without using de-noising techniques when the spectra of FBG1 and FBG2 are completely overlapped. As shown in Figure 12b, the proposed LSTM model measures the sensing signal of each FBG without using de-noising techniques when the spectra of the FBG1, FBG2, and FBG3 sensors are overlapped.

The sensing signal measurement performances of the proposed LTM model without using a DWT data de-noising method are 0.092 pm and 0.098 pm when two FBG spectra (test case a (Figure 12a)) and three FBGs spectra (test case b (Figure 12b)) are overlapped, respectively. On the other hand, Figure 13a,b shows the sensing signal measurement output of our proposed LSTM model in conjunction with using DWT data de-noising techniques when the spectra of two FBGs spectra and three FBGs spectra are overlapped, respectively.

As shown in Figure 13a, the proposed LSTM model accurately measured the sensing signals of FBGs using DWT data de-noising techniques even when the spectra of FBG1 and FBG2 are completely overlapped. As shown in Figure 13b, the proposed LSTM model in conjunction with using DWT de-noising techniques accurately measured the sensing signal of each FBGs even when the spectra of the FBG1, FBG2, and FBG3 sensors are overlapped. The sensing signal measurement performances of the proposed LTM model in conjunction with a DWT data de-noising method are 0.024 pm and 0.067 pm when the two FBG spectra (test case a (Figure 13a)) and three FBGs spectra (test case b (Figure 13b)) are overlapped, respectively.

The smaller the RMSE measurement error indicates that the measured value by our proposed model is nearly closer to the actual value. As a result, the experimental results, as shown in Figure 12, demonstrate that the sensing signal measurement performance using our proposed LSTM model without using a DWT is unsatisfactory. On the other hand, the performance of the LSTM model in conjunction with the DWT de-noising technique achieves a better performance even in noisy data, as shown in Figure 13. Hence, the LSTM model in conjunction with a DWT de-noising method is capable of efficiently improving the sensing signal measurement accuracy of the FBG sensor system. Therefore, our proposed deep learning algorithm proves that we can accurately measure the sensing signal of FBGs even with overlapped FBG spectra and noisy sensor data.

### 4.3. Performance Evaluation

Furthermore, to verify and validate the strain sensing measurement performance of our proposed deep learning model, we compare and contrast the performances of our proposed LSTM model with two other models: extreme learning machine (ELM) and multilayer perceptron (MLP). We computed the simulation using the same parameters, the same training and testing data, and under the same PC environment. For the ELM model, the number of hidden units is set to 1200. Table 1 indicates the comparison of strain sensing signal measurement performance of our proposed LSTM models with the other two models based on four different test cases. As shown in the table, the performances of our proposed LSTM model without using DWT method in test case a and test case b are 0.092 pm, and 0.098 pm, respectively.

On the other hand, the performances of our proposed LSTM model in conjunction with a DWT data de-noising method in test case i and test case ii are 0.024 pm and 0.067 pm, respectively. Hence, the RMSE of our proposed LSTM model is smaller than the other two models in all test cases. Therefore, our proposed LSTM model achieves better sensing signal measurement performance than the MLP and ELM models with a low-error sensing signal measurement. The LSTM algorithm has the ability to learn complex representations from the sequential features of the spectra of FBGs. The proposed LSTM model can also avoid the randomness and uncertainty of EAs with higher reliability. Thus, even when the adjacent FBG sensors spectra are fully or partly overlapped, the proposed LSTM model accurately measures the strain sensing signal of the four FBGs.

**Figure 14:**
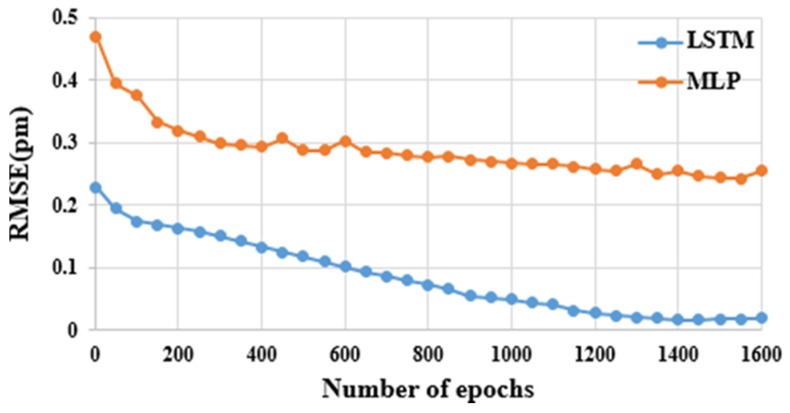
The performance comparisons of our proposed LSTM model with four different methods in different epoch numbers.

Furthermore, Figure 14 shows the performance comparison between our proposed LSTM models with an MLP model based on a different number of epochs.

In our proposed LSTM model, a small RMSE is achieved when the epoch number is 1400. Thus, as shown in the figure, even when the spectra of three FBG sensors are overlapped, the RMS errors are 0.024 pm and 0.258 pm for LSTM and MLP, respectively. As a result, our proposed LSTM model achieves better sensing signal measurement performance than MLP at different epoch numbers. The LSTM algorithm has the ability to learn complex representations from the sequential features of the spectra of FBGs.

**Figure 15:**
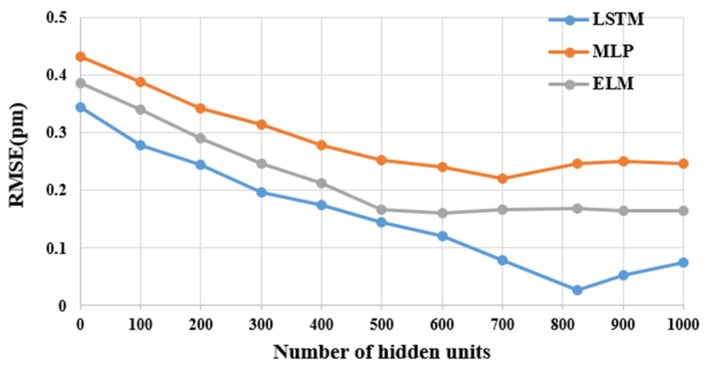
The performance comparisons of our proposed LSTM model with three different methods in different number of hidden unites.

Furthermore, the comparison of the RMS error from the proposed LSTM models with the other two models based on hidden unit numbers variation is shown in Figure 15. As shown in the figure, the RMS error decreases as the numbers of hidden units increases and LSTM has better performance than the other two models. Our proposed LSTM model achieves a better result when the hidden unit is 824. Therefore, the LSTM-based strain sensing signal measurement method can improve the sensing signal measurement accuracy, speed, and the number of FBG sensors even in bad or noisy environments.

## 5. Conclusions

In this paper, we proposed an LSTM integrated with data de-noising techniques for an IWDM-based multipoint FBG sensor system to improve the sensing signal measurement accuracy. As the performance of our proposed LSTM model depends on the sensing signal measurement of FBGs, we calculated the sensing signal measurement errors to test the effectiveness of our proposed LSTM model. First, we used DWT data de-noising methods to reduce the noise and processed the noisy FBG signals. Then, we utilized our proposed LSTM model to measure the sensing signal of each FBG from the de-noising FBG signal. The well-trained LSTM model learned from the sequential features of the FBG sensors spectra and can identify the strain sensing signal of FBGs for the entire FBG sensor system.

A significant benefit of the proposed LSTM model is that, in any bad environment or with noisy sensor data, we do not require retraining or building of a new model. As a result, the well-trained LSTM model achieves a better sensing signal measurement performance even though the spectra of FBG sensors completely overlap and the sensor data are noisy. Compared with other traditional machine learning techniques, the LSTM model achieves a high sensing signal measurement accuracy performance. Therefore, the proposed LSTM model can increase the number of FBG sensors in the sensor system and improves the sensing signal measurement accuracy performance of the IWDM FBG sensor network.

## Figures and Tables

**Table 1:** The performance comparison results of four different models in four different testing cases.

		RMSE (pm)		
	Test Case	LSTM	MLP	ELM
Without using DWT de-noising techniques	a (Figure 12a)	0.092	0.237	0.175
	b (Figure 12b)	0.098	0.274	0.207
With using DWT de-noising techniques	i (Figure 13a)	0.024	0.243	0.168
	ii (Figure 13b)	0.067	0.258	0.200
